# Chlorogenic Acid as a Modulator of Adipose Tissue Function and Metabolic Homeostasis: Evidence from Preclinical Studies

**DOI:** 10.3390/nu18142358

**Published:** 2026-07-18

**Authors:** Arshpreet Sehra, Evangelia Tsiani

**Affiliations:** 1 Department of Health Sciences, Brock University, St. Catharines, ON L2S 3A1, Canada; 2 Centre for Bone and Muscle Health, Brock University, St. Catharines, ON L2S 3A1, Canada

**Keywords:** chlorogenic acid, adipogenesis, thermogenesis, insulin signaling

## Abstract

Obesity is an escalating global health challenge, driven by adipose tissue dysfunction characterized by adipocyte hypertrophy, chronic low-grade inflammation and impaired insulin signaling, which together promote insulin resistance, dyslipidemia, hepatic steatosis and cardiovascular disease. Chlorogenic acid (CGA), a dietary phenolic phytochemical abundant in coffee, tea, fruits and vegetables, has attracted considerable interest as a modulator of adipocyte biology and metabolism. This review summarizes in vitro and in vivo evidence of the effects of CGA on adipogenesis, lipid metabolism, thermogenesis, inflammation and glucose homeostasis. In vitro studies employing murine and human adipocyte and progenitor cell models demonstrate that CGA attenuates adipocyte differentiation and lipid accumulation via downregulation of adipogenic transcription factors (PPARγ, C/EBPα) and lipogenic enzymes (FASN, ACC, SREBP 1c), alongside activation of AMPK, Shp2–Erk1/2 and Wnt–β catenin signaling. Several reports further show that CGA promotes browning of white adipocytes and enhances thermogenic and mitochondrial gene expression. Complementary in vivo studies in diet-induced obesity and diabetes models reveal that CGA reduces body weight gain, adiposity, adipocyte size and hepatic steatosis, improves lipid profiles, glucose tolerance and insulin sensitivity, and exerts anti-inflammatory and antioxidant effects in metabolic tissues. Collectively, current preclinical evidence supports CGA as a multifaceted modulator of adipose tissue function and whole-body metabolic homeostasis; however, rigorously designed, long-term clinical trials are required to establish its efficacy and safety in humans.

## 1. Introduction

Obesity has emerged as one of the most significant public health challenges of the 21st century, affecting more than 1 billion individuals worldwide and imposing substantial burdens on healthcare systems and economies globally. According to recent estimates from the NCD Risk Factor Collaboration, obesity prevalence among adults more than doubled between 1990 and 2022, while rates among children and adolescents (5–19 years) quadrupled during this period [1]. In 2022, approximately 16% of adults worldwide were living with obesity, and by 2035, this figure is projected to rise to 1.53 billion, with 79% of affected adults residing in low- and middle-income countries [2]. Beyond direct medical costs, obesity contributes substantially to lost productivity, increased disability-adjusted life years, and premature mortality, undermining the urgent need for effective prevention and treatment strategies.

Adipocytes, the principal cellular constituents of adipose tissue, serve critical roles in energy storage, thermogenesis, and endocrine signaling through secretion of adipokines, cytokines, and metabolic mediators [3,4]. In obesity, adipose tissue undergoes pathological remodeling, which is characterized by adipocyte hypertrophy, hypoxia, chronic low-grade inflammation, macrophage infiltration, and impaired insulin signaling promoting systemic insulin resistance, dyslipidemia, hepatic steatosis, and increased cardiovascular disease risk [5,6,7,8]. Notably, adipose tissue dysfunction manifests across diverse metabolic disorders, from obesity-associated insulin resistance to type 2 diabetes, highlighting that proper adipocyte function is essential for metabolic homeostasis [5]. Understanding in detail the molecular mechanisms involved in adipocyte differentiation, lipid metabolism, and inflammatory responses is therefore critical for developing targeted therapeutic interventions.

**Figure 1:**
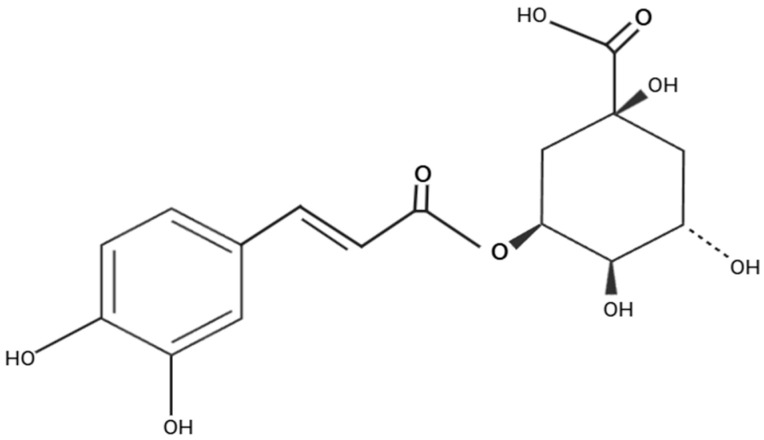
Chemical structure of chlorogenic acid (5-O-caffeoylquinic acid). The figure was created using https://BioRender.com (accessed on 16 May 2026).

Chlorogenic acid (CGA), a phenolic phytochemical formed by esterification of caffeic acid (CA) with L-quinic acid, represents one of the most abundant and widely distributed polyphenolic compounds in the human diet (Figure 1) [9]. Coffee represents the predominant dietary source of CGA, with a single serving providing 20–675 mg depending on roast type and preparation method. Other significant dietary sources include green tea, apples, blueberries, tomatoes, and potatoes [10]. Structurally, CGA exists in different isomeric forms, with 5-O-caffeoylquinic acid being the most prevalent isoform. The molecule contains three chemically labile groups—an ester bond, an unsaturated double bond, and polyphenolic hydroxyl groups—which contribute to its biological activities and metabolic effects [11,12,13]. CGA has been implicated in a wide range of biological functions, including antioxidant [14,15], neuroprotective [15,16,17], anti-cancer [18], anti-inflammatory [19], and anti-diabetic functions [9,20].

A few reviews have examined the metabolic benefits of chlorogenic acid (CGA) [9,20] and its anti-obesity effects [21,22]. However, previous reviews have primarily examined the effects of CGA as a constituent of plant extracts rather than as an isolated compound. Consequently, it is difficult to attribute the reported biological effects specifically to CGA, as other bioactive constituents present in the extracts may have contributed to the observed outcomes. Furthermore, previous reviews have not evaluated the effects of CGA in combination with other therapeutic agents, representing another important gap in the current literature. Furthermore, existing reviews lack a comprehensive mechanistic framework to explain the specific actions of CGA within adipose tissue. In addition, many do not critically evaluate the strength and limitations of the available evidence or adequately identify current knowledge gaps and priorities for future research.

The present review addresses these limitations by synthesizing evidence from both in vitro and in vivo studies investigating the effects of chlorogenic acid on adipocyte function and obesity-related outcomes, while providing a comprehensive overview of the underlying molecular mechanisms. A literature search was conducted using PubMed with the following keywords: “chlorogenic acid and obesity,” “chlorogenic acid and adipocytes,” “chlorogenic acid and adipose tissue,” “chlorogenic acid and brown adipocytes,” “chlorogenic acid and beige adipocytes,” “chlorogenic acid and functional brown adipose tissue,” and “chlorogenic acid and insulin resistance and adipocytes.” Articles were excluded if they were not directly relevant to the review topic or were unavailable in English.

## 2. The Effects of CGA on Adipose Tissue

### 2.1. In Vitro Evidence

Zhou et al. (2015) [23] treated bone marrow-derived mesenchymal stem cells (BMSCs) with CGA for 14 days, followed by Oil Red O staining [23]. CGA significantly inhibited lipid accumulation under adipogenic differentiation conditions compared to adipogenic medium alone, and this anti-adipogenic effect was reversed by the Shp2 inhibitor NSC87877 (10 μM) and the Erk inhibitor PD98059 (20 μM), demonstrating Shp2/Erk pathway dependence. RT-qPCR analysis demonstrated that CGA dose-dependently decreased mRNA levels of the master adipogenic transcription factors PPARγ and C/EBPα and significantly enhanced Erk1/2 phosphorylation (Table 1). Shp2 knockdown using siRNA abolished CGA’s ability to suppress adipogenesis, further indicating that Shp2 was essential for CGA’s anti-adipogenic effects [23]. Collectively, these findings demonstrate that CGA inhibited adipogenic differentiation of BMSCs by activating Shp2-mediated Erk1/2 signaling, which subsequently suppressed PPARγ and C/EBPα expression, providing a novel mechanism by which CGA prevents adipogenesis in stem cell populations.

Sanchez et al. (2017) [24] differentiated 3T3-L1 preadipocytes into mature adipocytes and treated them with CGA (50 μM) or pioglitazone (10 μM) as positive controls for 24 h. Real-time PCR analysis of differentiated 3T3-L1 adipocytes revealed that CGA treatment (50 μM) significantly upregulated PPARγ mRNA to levels similar to those achieved with pioglitazone. CGA treatment dramatically increased GLUT4 and PPARα mRNA levels compared to control. CGA treatment also increased fatty acid transport protein (FATP) mRNA levels. Molecular docking analysis using Swiss ADME, Molinspiration, and Osiris servers indicated that CGA possesses favorable physicochemical properties and has a 74% probability of functioning as a nuclear receptor ligand, supporting its mechanism as a PPAR agonist [24]. Although these findings demonstrate that CGA may function as a dual PPAR-α/γ agonist in adipocytes, suggesting therapeutic potential for treating metabolic diseases through enhanced insulin sensitivity and lipid oxidation, the data are limited. It would have been more convincing if the researchers examined the protein levels as well.

Han et al. (2019) [25] cultured C3H10T1/2 mouse embryonic fibroblasts, which underwent brown adipogenic differentiation with CGA for 6 days. CGA did not alter adipogenic markers (C/EBPα, PPARγ2, PRDM16) or lipid droplet accumulation, as confirmed by Oil Red O staining. However, CGA significantly upregulated UCP1 mRNA and protein, while also increasing PGC1α mRNA levels. CGA did not upregulate fatty acid oxidation genes (PPARα, CPT1α, ATGL, HSL, ACS); however, cell respirometry demonstrated enhanced proton leak and reduced ATP production. CGA promoted glucose consumption and significantly upregulated GLUT2 and phosphofructokinase (PFK) expression. Western blot demonstrated significant increases in TFAM, OXPHOS proteins (ATP5A, UQCRC2, SDHB), and p-AMPK, while mtDNA copy number was significantly elevated [25]. Collectively, these findings demonstrate that CGA enhanced thermogenesis in brown adipocytes through AMPK-dependent promotion of glucose uptake and mitochondrial function, independent of adipocyte differentiation and fatty acid oxidation.

Treatment of 3T3-L1 adipocytes with CGA significantly reduced lipid droplet accumulation. Real-time PCR and Western blot analyses revealed that CGA dose-dependently downregulated PPAR-γ and its target genes (aP2, FAS, LPL) at both mRNA and protein levels. CGA treatment increased β-catenin and Wnt10b protein levels (Table 1). Immunofluorescence demonstrated that CGA promoted cytosolic β-catenin accumulation and nuclear translocation. CGA inhibited total GSK-3β expression while inducing GSK-3β phosphorylation [26]. Collectively, these findings demonstrate that CGA inhibited adipogenesis through activation of the Wnt/β-catenin signaling pathway by inducing GSK-3β phosphorylation, stabilizing β-catenin, and downregulating PPAR-γ and downstream adipogenic genes.

**Figure 2:**
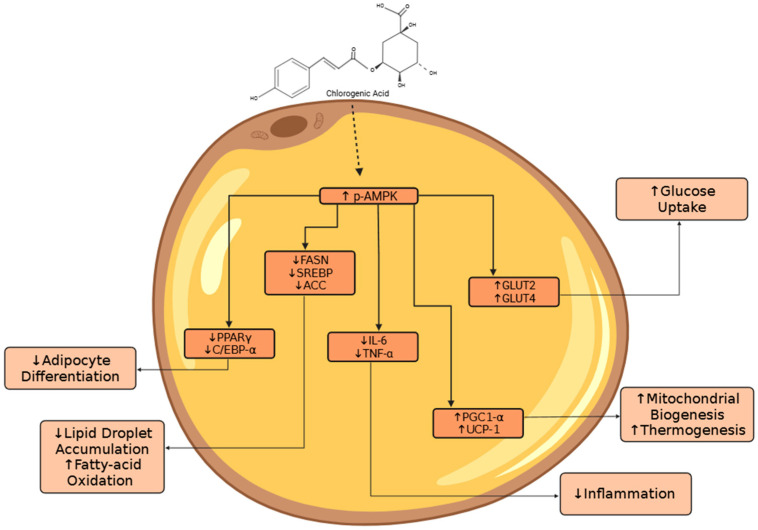
Summary of the effects of CGA in adipocytes from in vitro studies. The figure was created using https://BioRender.com (accessed on 16 May 2026).↑: increased, ↓: decreased, p: phosphorylated.

Molonia et al. (2026) [27] subjected 3T3-L1 adipocytes to repeated H_2_O_2_ (100 µM) exposure during the differentiation process (days 5–7) to induce a senescent phenotype, followed by treatment with CGA (5–20 µM) until day 10 [27]. CGA dose-dependently reduced senescence-associated β-galactosidase (SA-β-Gal) activity and restored Lamin B1 protein levels, while attenuating the activation of the p53/p21 cell cycle checkpoint axis and MAPK pathway members (Phospho-p38, pERK1/2), consistent with a reprogramming of senescence-associated cell cycle arrest. Moreover, CGA restored the Bcl-2/BAX ratio toward control values, counteracting the apoptotic resistance characteristic of the senescent phenotype, and simultaneously attenuated intracellular ROS accumulation and nuclear NF-κB (p65) translocation, resulting in the downregulation of key SASP-associated mediators, including IL-6, IL-8, TNF-α, MMP-3, and COX-2. Notably, CGA dose-dependently rescued insulin signaling through the PI3K–AKT–GLUT4 axis, significantly improving glucose uptake in senescent adipocytes, and preserved adipogenic capacity, as evidenced by the restoration of PPARγ protein levels, FASN mRNA levels, and intracellular lipid accumulation assessed by Oil Red O staining. These data suggest that CGA simultaneously targets oxidative stress, SASP-associated inflammatory signaling, insulin resistance, and impaired adipogenesis, thereby representing a promising dietary nutraceutical candidate for counteracting age-related adipose tissue dysfunction and its associated metabolic consequences (Figure 2).

### 2.2. In Vivo Evidence

Cho et al. (2010) [28] administered CGA (0.02% *w*/*w*, approximately 0.2 g/kg diet) to male ICR mice consuming a high-fat diet (HFD; 37% calories from fat; 21% beef tallow) for 8 weeks [28]. CGA significantly reduced final body weight by 16% and body weight gain compared to HFD-fed mice, without affecting food intake or daily energy intake. CGA significantly decreased eWAT weight by 46% and perirenal adipose tissue weight by 58% compared to HFD control mice. CGA significantly reduced plasma triglycerides, total cholesterol, and free fatty acid levels, while increasing the HDL-cholesterol to total cholesterol ratio. CGA significantly lowered plasma leptin and insulin levels compared to HFD mice, and significantly increased plasma adiponectin levels (the only group among treatments to do so). CGA significantly reduced triglyceride concentrations in liver and heart, and adipose tissue triglyceride content, while reducing cholesterol levels in adipose tissue and heart. In the liver, CGA significantly inhibited fatty acid synthase (FAS), HMG-CoA reductase, and ACAT activities, while significantly increasing fatty acid β-oxidation activity to levels similar to normal diet-fed mice (Table 2). CGA significantly increased hepatic PPARα expression compared to HFD control mice. Body weight was significantly positively correlated with plasma leptin (r = 0.894, *p* < 0.01) and insulin (r = 0.496, *p* < 0.01) levels. Collectively, these findings suggest that CGA consumption prevents weight gain while simultaneously suppressing fatty acid and cholesterol biosynthesis and enhancing fatty acid oxidation in the HFD mouse models.

Ma et al. (2015) [29] utilized male C57BL/6J mice to examine the effects of CGA (CGA) on high-fat diet (HFD)-induced obesity, hepatic steatosis, and insulin resistance using both preventive and therapeutic protocols. In the prevention protocol, 6-week-old mice fed HFD (60% kcal from fat) for 15 weeks received intraperitoneal CGA (100 mg/kg, twice weekly) or vehicle from the onset of HFD feeding, while in the treatment protocol, diet-induced obese mice maintained on HFD for 15 weeks were subsequently treated with CGA for 6 weeks. In the prevention protocol, CGA markedly attenuated HFD-induced weight gain (39.0 ± 1.8 g vs. 51.2 ± 2.1 g in HFD controls) and adiposity without altering food intake, prevented adipocyte hypertrophy in eWAT and brown adipose tissue (BAT), and suppressed adipose tissue inflammation by reducing macrophage infiltration markers (F4/80, CD68, CD11b, CD11c) and pro-inflammatory cytokines (TNFα, MCP-1, CCR2). CGA conferred robust protection against hepatic steatosis, with reduced liver weight, hepatic triglyceride and cholesterol content, suppressed hepatic expression of PPARγ and its lipid-uptake genes (CD36, FABP4, MGAT1), and upregulated fatty acid oxidation genes (CPT1a, CPT1b, PPARα, ACOX1, FGF21). CGA also improved glucose homeostasis and insulin sensitivity, reducing fasting blood glucose (134 ± 28 vs. 204 ± 43 mg/dL), insulin levels (0.7 vs. 5.3 µg/L), HOMA-IR indices, and pancreatic Insulin1/Insulin2 expression, while enhancing glucose clearance and insulin responsiveness [29]. In the treatment protocol, CGA administered to established obese mice did not reduce body weight but significantly improved glucose tolerance/insulin sensitivity, reducing hepatic steatosis while enhancing PPARγ signaling and fatty acid oxidation. Collectively, these findings indicate that CGA prevents diet-induced obesity and metabolic syndrome, improves insulin sensitivity and reduces hepatic steatosis in established obesity through inhibition of hepatic PPARγ signaling, enhancement of lipid oxidation, and suppression of adipose tissue inflammation.

Jin et al. (2015) [30] administered CGA (80 mg/kg/day) or vehicle (PBS) via oral gavage to female C57BL/BKS db/db diabetic mice for 12 weeks. CGA significantly reduced the percentage of visceral adipose tissue (VA), fasting plasma glucose (FPG), and glycosylated hemoglobin (HbA1c) compared to db/db control mice, without affecting food intake or plasma triglycerides, total cholesterol, or insulin levels. CGA significantly increased adiponectin levels in visceral adipose tissue and decreased visfatin levels in db/db mice. CGA significantly reduced aldose reductase (AR) activity in the kidney and transforming growth factor-β1 (TGF-β1) protein levels, indicating improved diabetic nephropathy in db/db mice (Table 2). In liver tissue, CGA significantly upregulated adiponectin receptor 2 (ADPNR-2) expression, increased phosphorylated AMPK (p-AMPK) levels, and downregulated glucose-6-phosphatase (G-6-P) protein levels. CGA significantly upregulated hepatic PPAR-α mRNA and protein levels. In skeletal muscle, CGA significantly increased ADPNR-1 protein levels, p-AMPK levels, and GLUT-4 protein levels [30]. Collectively, these findings demonstrate that CGA ameliorated late-stage diabetes in db/db mice by improving insulin sensitivity and glucose homeostasis through adiponectin receptor-mediated signaling pathways involving AMPK phosphorylation and PPAR-α upregulation, while also improving diabetic kidney fibrosis through reduced AR activity and TGF-β1 expression.

Zhou et al. (2016) [31] administered CGA (60 mg/kg body weight/day) to female Sprague-Dawley rats for 28 days, with and without chronic low-dose endotoxin infusion (300 µg/kg body weight/day intraperitoneally) to induce lipid metabolic disorder. CGA significantly reduced body weight gain in the lipid dysregulated (LD) rats during weeks 3–4 and total body weight gain without affecting food intake. CGA significantly reduced visceral adipose tissue weight and decreased adipocyte area compared to LD rats, as demonstrated by H&E histology. CGA significantly reduced serum triglycerides and free fatty acids, and increased HDL-cholesterol compared to LD rats, while total cholesterol and LDL-cholesterol were not significantly affected. CGA significantly ameliorated endotoxin-induced liver injury, reducing serum bilirubin, ALT, and AST activity, and decreasing hepatic triglycerides and cholesterol content, as confirmed by Oil Red O staining showing reduced lipid droplet accumulation. In liver tissue, CGA significantly increased fatty acid β-oxidation activity, CPT-1 content, and CPT-1 activity, while significantly decreasing ACC content and FASN content and activity. CGA significantly increased phosphorylated AMPK (p-AMPK) levels in liver tissue. CGA modulated hepatic fatty acid composition by decreasing desaturase activity indexes and shifting the profile toward protective fatty acids [31]. Collectively, these findings demonstrate that CGA effectively ameliorated endotoxin-induced lipid dysregulation by activating AMPK, enhancing fatty acid β-oxidation, suppressing fatty acid and cholesterol synthesis, and modulating hepatic fatty acid composition.

Wang et al. (2019) [32] administered CGA (150 mg/kg/day) to male C57BL/6J mice consuming a high-fat diet (HFD; 18.4% fat) for 6 weeks. CGA significantly reduced body weight, weight gain, liver weight, and eWAT weight compared to HFD-fed mice. Histological examination revealed that CGA treatment greatly attenuated fat mass and adipocyte size in eWAT and reduced hepatic steatosis by decreasing abnormal lipid droplet accumulation. CGA significantly reduced plasma triglycerides, total cholesterol, and LDL-cholesterol levels while increasing HDL-cholesterol compared to HFD controls. Plasma ALT, AST, and BUN levels elevated in HFD mice were significantly suppressed by CGA treatment, indicating improved hepatic and renal function. In eWAT, CGA significantly downregulated mRNA expression of adipogenic and lipogenic markers (PPAR-γ, C/EBP-α, SREBP-1c, FAS, LPL, AP2, and GRP43), while significantly upregulating PPARα and adiponectin mRNA expression. CGA treatment dramatically reversed HFD-induced gut microbiota dysbiosis by significantly inhibiting the growth of Desulfovibrionaceae, Ruminococcaceae, Lachnospiraceae, and Erysipelotrichaceae, while promoting the growth of Bacteroidaceae and Lactobacillaceae [32]. Collectively, these findings suggest that CGA exerted anti-obesity and hypolipidemic effects through regulation of lipogenesis and lipolysis genes in white adipose tissue, with improvements in gut microbiota composition contributing to its beneficial metabolic effects.

Ye et al. (2021) [33] administered CGA (150 mg/kg body weight/day) or vehicle via oral gavage to male C57BL/6 mice for 20 weeks, with half receiving a normal diet (NFD) and half a high-fat diet (HFD). CGA significantly inhibited HFD-induced body weight gain without affecting food intake and substantially reduced subcutaneous (inguinal), perirenal, and eWAT weights. CGA significantly improved glucose homeostasis by reducing fasting blood glucose, fasting insulin levels, and HOMA-IR index, with enhanced insulin sensitivity and glucose tolerance on both OGTT and ITT. CGA significantly reduced plasma LPS (lipopolysaccharide) levels and decreased serum TNF-α, IL-1β, and MCP-1 concentrations in HFD-fed mice. CGA inhibited TLR-4 expression in liver and eWAT and improved intestinal barrier integrity by increasing colon length, reducing plasma FITC-dextran levels, and upregulating mRNA and protein of tight-junction proteins (ZO-1, claudin-1, occludin) in the ileum (Table 2). At the phylum level, CGA shifted the gut microbiota by decreasing HFD-induced increases in Firmicutes while increasing Bacteroidetes and Verrucomicrobia abundance, reducing the elevated Firmicutes/Bacteroidetes ratio. At the family level, CGA decreased Lachnospiraceae and Erysipelotrichaceae while increasing Muribaculaceae and Akkermansiaceae abundance. At the genus level, CGA significantly increased SCFA-producing bacteria (*Dubosiella*, *Romboutsia*, *Mucispirillum*, *Faecalibaculum*) and *Akkermansia*, which protects intestinal barrier function. Fecal microbiota transplantation from CGA-treated mice to HFD-fed recipients resulted in decreased body weight, adipose tissue accumulation, liver weight, improved glucose tolerance and insulin sensitivity, and reduced plasma LPS and FITC-dextran levels [33]. Collectively, these findings demonstrate that CGA-induced alterations in gut microbiota composition, particularly enrichment of SCFA-producing bacteria and Akkermansia, were the primary mechanism by which CGA inhibited HFD-induced metabolic endotoxemia, low-grade inflammation, and obesity through improvements in intestinal barrier integrity.

Yan et al. (2022) [34] administered CGA (0.25 g/kg body weight/day) or metformin (0.25 g/kg body weight/day) via oral gavage to male db/db diabetic mice for 18 days. CGA significantly reduced body weight gain and body weight compared to untreated db/db mice (Day 18: 37.93 g vs. 41.30 g) and lowered daily feed intake without affecting overall metabolic rate. CGA significantly decreased inguinal fat weight by 23% and liver weight by 6% compared to db/db control mice, though epididymal adipose tissue weight was not significantly altered. CGA significantly improved glucose tolerance and insulin sensitivity and reduced fasting blood glucose. CGA significantly lowered serum triglycerides (1.23 mM vs. 2.02 mM), total cholesterol, LDL-cholesterol, while increasing HDL-cholesterol; serum ALT and AST were significantly reduced (43.1 U/L and 28.3 U/L respectively). Hepatic histological examination (H&E and Oil Red O staining) confirmed that CGA decreased lipid accumulation and hepatocyte damage. In liver tissue, CGA significantly upregulated mRNA expression of genes involved in fatty acid oxidation (CPT1a, ACOX1), lipolysis (ATGL, HSL), and anti-inflammatory/antioxidant genes (IL-10, SOD1, SOD2, GPX1), while significantly downregulating genes involved in triglyceride synthesis (MGAT1, DGAT2) and fatty acid transport (CD36, FATP4). CGA significantly downregulated pro-inflammatory genes (TNF-α, IL-1β, IL-6). CGA improved cecal microbiota alpha and beta diversity, recovered the abundance of beneficial bacteria (Bacteroidetes and *Lactobacillus*), and reduced pathogenic bacteria (*Blautia*, *Robinsoniella*, *Enterococcus*) [34]. Collectively, these findings demonstrate that acute CGA administration ameliorates hyperglycemia, hepatic lipid accumulation, and inflammation in db/db mice through enhanced fatty acid oxidation and lipolysis, reduced lipogenic gene expression, and modulation of gut microbiota composition, with effects comparable to metformin.

**Figure 3:**
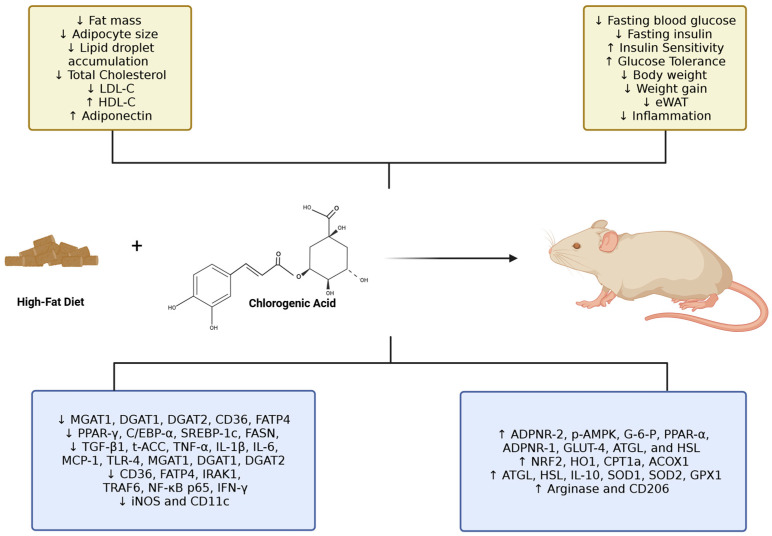
Summary of the effects of CGA in adipocytes from in vivo studies. The figure was created using https://BioRender.com (accessed on 16 May 2026).↑: increased, ↓: decreased, p: phosphorylated, t: total.

Alenezi et al. (2025) [35] administered CGA (10 mg and 100 mg/kg/day) to male Sprague-Dawley rats for the last 4 weeks of a 12-week high-fat diet (HFD) protocol. CGA significantly reduced body weight gain, BMI, abdominal circumference, and abdominal WAT mass. CGA significantly improved metabolic hormone levels by reducing serum insulin, fasting blood glucose, HOMA-IR, and leptin, while increasing adiponectin. CGA significantly improved lipid homeostasis by reducing total cholesterol, triglycerides, and LDL-c, while increasing HDL-c. CGA significantly attenuated hepatic dysfunction (reduced ALT and AST) and renal dysfunction (reduced creatinine and BUN). CGA significantly improved oxidative stress markers by restoring TAC, reducing MDA, and altering inflammatory cytokines (reduced IL-1β and TNF-α, increased IL-10). CGA significantly affected hypothalamic appetite-regulating gene expression by downregulating Agrp and NPY mRNA, while upregulating POMC and CARTPT mRNA. In abdominal WAT, CGA significantly upregulated miR-146a expression while reducing its downstream targets IRAK1 and TRAF6, as well as TNF-α, NF-κB p65, IL-1β, and IFN-γ mRNA, while partially restoring IL-10 mRNA (Figure 3). CGA significantly altered apoptotic pathways by reducing p53, Bax, and Caspase-3 mRNA, while restoring Bcl-2 mRNA. CGA significantly enhanced antioxidant defenses by upregulating NRF2, HO1, SOD, CAT, and GPx mRNA expression in WAT [35]. Collectively, these findings suggest the potential of CGA to attenuate obesity-related metabolic disorders through anti-inflammatory, antioxidant, and appetite-regulatory mechanisms.

### 2.3. Evidence from Combination Studies

Vasileva et al. (2020) [36] cultured human Simpson–Golabi–Behmel syndrome (SGBS) preadipocytes, which were exposed to caffeic acid (CA) and CGA at concentrations of 5, 10, and 50 µM for 9 days during white adipogenic differentiation. Dose-dependent decreases in intracellular lipid accumulation were observed through Oil Red O staining, with significant reductions observed at all concentrations (*p* < 0.05). Real-time PCR revealed that CA/CGA co-treatment robustly upregulated AMPK mRNA to levels exceeding individual treatments. Brown adipocyte markers UCP1 and PGC1α were significantly upregulated at lower concentrations, with hierarchical cluster analysis revealing that gene expression profiles resembled brown-like adipocyte signatures. Lipogenic markers ACC, FASN, and SREBP1 were significantly downregulated (*p* < 0.05) (Table 3). Western blot demonstrated dose-dependent reductions in adiponectin, C/EBPα, and PPARγ, with the CA/CGA combination at 5 µM significantly activating PPARγ to levels nearly equal to 50 µM CGA alone (*p* < 0.05) [36]. Collectively, these findings indicate that CA/CGA co-treatment exerted browning-inducing potential through AMPK and PPAR-dependent pathways.

Kong et al. (2021) [37] cultured 3T3-L1 preadipocytes, which underwent differentiation with exposure to CGA (40 μg/mL) and caffeine (160 μg/mL) beginning at Day 0 for 4 days. Both compounds demonstrated non-significant cytotoxicity at the selected concentrations by MTT assay, with CGA + caffeine showing no cytotoxicity to 3T3-L1 cells. According to triglyceride (TG) assay and Oil Red O staining performed at Day 12, the CGA + caffeine combination markedly reduced TG content compared to control, with CGA + caffeine (*p* < 0.01) and CGA alone (*p* < 0.05) showing obvious effects on TG levels, though caffeine alone had no effect; moreover, CGA + caffeine demonstrated greater inhibition of lipid accumulation than CGA used individually. At Days 8, 10, and 12, real-time quantitative PCR analysis revealed that CGA + caffeine increased mRNA expression of AMPK, ACO, CAT, ATGL, GLUT4, and HSL while decreasing mRNA expression of GPDH, with significant differences particularly evident at Day 12 (*p* < 0.05). Examination of transcription factor expression during differentiation showed that CGA + caffeine most potently inhibited mRNA expression of PPAR-γ2 and C/EBPα at Days 2 (metaphase) and 4 (anaphase) of differentiation, suggesting inhibition of adipocyte differentiation occurs predominantly during these middle and late stages. At Day 12, enzyme activity analysis demonstrated that CGA and CGA + caffeine inhibited GPDH and FASN activities while enhancing HSL activity, thereby reducing lipid deposition and promoting lipid hydrolysis. Western blot analysis at Day 12 revealed that CGA + caffeine upregulated protein levels of phosphorylated AMPK/total AMPK, ATGL, and phosphorylated HSL/total HSL while downregulating FASN protein levels [37]. Collectively, these findings demonstrate that CGA + caffeine attenuated adipogenesis and reduced fat accumulation in 3T3-L1 cells through AMPK pathway activation, with the combination displaying stronger anti-adipogenic effects than either compound administered alone, mediated by inhibition of adipocyte differentiation during metaphase and anaphase stages coupled with suppression of lipid synthesis and enhancement of lipolysis and fatty acid oxidation.

Xu et al. (2019) [38] administered CGA and CF alone or in combination to female ICR mice consuming a high-fat diet (HFD; 45% calories from fat) for 14 weeks. The combination of 0.2% CGA + 0.02% CAF and 0.2% CGA + 0.04% CAF significantly reduced body weight gain and intraperitoneal adipose tissue (IPAT) weight compared to HFD controls, with effects comparable to caffeine (0.04%) alone but requiring lower caffeine doses when combined with CGA. CGA + CAF combination significantly reduced serum total cholesterol, triglycerides, LDL-cholesterol, free fatty acids, leptin, and IL-6 concentrations, while significantly increasing serum adiponectin levels compared to HFD controls. CGA + CAF combination significantly decreased hepatic triglycerides and cholesterol content, with histological analysis showing substantially reduced lipid droplet accumulation in liver tissue. In hepatic tissue, the CGA + CAF combination significantly upregulated mRNA expression of AMPKα, ATGL, ACO, and HMGR, while significantly downregulating FAS, SCD1, SREBP-1c, and LXRα mRNA expression. CGA + CAF combination significantly increased protein levels of phosphorylated AMPKα (p-AMPKα), ATGL, and HSL, while significantly decreasing the protein levels of FAS, SCD1, and LXRα in the liver (Table 3). Fecal lipid analysis revealed that the CGA + CAF combination increased fecal triglyceride and cholesterol excretion, indicating enhanced lipid elimination. There was no significant difference in energy intake between HFD and treatment groups [38]. Collectively, these findings demonstrate that the combination of CGA and caffeine synergistically regulated lipid metabolism through the AMPKα-LXRα/SREBP-1c signaling pathway, enhancing fatty acid β-oxidation and lipolysis while suppressing fatty acid and cholesterol synthesis, with CGA permitting reduced caffeine doses to achieve anti-obesity effects comparable to caffeine alone.

Khalafani et al. (2023) [39] administered metformin (MET) (0.25% *w*/*w* in diet), CGA (0.02% *w*/*w* in diet), or their combination to male C57BL/6 mice consuming a high-fat diet (HFD) for 10 weeks, with normal diet (ND) controls. MET and CGA, alone or in combination, significantly reduced body weight gain and plasma triglyceride, glucose, and insulin levels compared to HFD controls. The combination of MET + CGA was particularly effective at reducing weight gain, with effects superior to either agent alone. MET + CGA combination significantly attenuated skeletal muscle (SM) inflammation, an effect associated with decreasing macrophage infiltration rate into the muscle tissue. Combined treatment of MET + CGA resulted in switching macrophages from pro-inflammatory M1 phenotype to anti-inflammatory M2 phenotype, as demonstrated by higher expression levels of M2 markers (arginase and CD206) and lower expression levels of M1 markers (iNOS and CD11c). The combination treatment was more effective than single treatments in increasing anti-inflammatory cytokine expression (IL-10) while decreasing pro-inflammatory mediator expression (TNF-α, IL-1β, MCP-1, and IL-6) in skeletal muscle tissue (Table 3). The combination treatment resulted in significantly lower fasting blood glucose levels and improved glucose tolerance compared to HFD controls [39]. Collectively, these findings suggest that the combination treatment of MET and CGA represents a promising therapeutic approach for controlling skeletal muscle inflammation and metabolic dysfunction in high-fat diet-induced obesity.

### 2.4. Evidence from Human Studies

Thom (2007) [40] conducted two sequential clinical studies to evaluate the effect of chlorogenic acid (CGA)-enriched instant coffee (Coffee Slender^®^, containing 90–100 mg CGA via Svetol^®^ green coffee extract per sachet) on glucose absorption and body composition [40]. In Study 1, a three-way double-blind randomized crossover trial in 12 healthy volunteers (mean age 24.2 ± 3.2 years, BMI < 25 kg/m^2^), consumption of CGA-enriched coffee alongside a 25 g sucrose load significantly reduced the glucose area under the curve (AUC), an effect not observed with regular caffeinated or decaffeinated instant coffee, demonstrating specificity to CGA rather than caffeine. In Study 2, a 12-week randomized placebo-controlled trial in 30 overweight volunteers (BMI 27.5–32.0 kg/m^2^), participants consuming five cups/day of CGA-enriched coffee lost a mean of 5.4 ± 0.6 kg compared with 1.7 ± 0.9 kg in the regular coffee group; notably, approximately 80% of the weight reduction in the CGA group was attributable to fat loss, as evidenced by a significant decrease in body fat percentage from 27.2 ± 2.0% to 23.6 ± 1.7%, with no significant fat loss in the control group. The authors attributed these effects to CGA’s capacity to inhibit intestinal glucose absorption via disruption of the Na^+^ electrochemical gradient in enterocytes and to suppress hepatic glucose-6-phosphatase activity, thereby reducing postprandial insulinemia and redirecting metabolism toward fat oxidation.

Soga et al. (2013) [41] conducted a placebo-controlled, double-blind, randomized crossover intervention study in 18 healthy male subjects (mean age 36.1 ± 7.4 years) to investigate whether daily chlorogenic acid (CGA) consumption influences energy metabolism in humans [41]. Subjects consumed 185 mL of either a CGA-containing beverage (329 mg CGAs/can, comprising nine CGA isomers including caffeoylquinic and feruloylquinic acids) or a matched control beverage (0 mg CGAs) daily for two 4-week intervention periods separated by a 3-week washout, with energy metabolism assessed by indirect calorimetry during fasting and up to 180 min postprandially at the start and end of each period. After 4 weeks, subjects in the CGA group exhibited significantly higher postprandial energy expenditure than controls, and postprandial fat utilization was significantly elevated in the CGA group relative to the initial value, while carbohydrate utilization and body composition were unchanged between groups (Table 4). Fasting blood glucose was also significantly reduced in the CGA group after the intervention. The authors proposed that these effects may be mediated, at least in part, through CGA-induced suppression of SREBP-1c—a master transcriptional regulator of lipogenesis—leading to increased mitochondrial fatty acid oxidation, consistent with prior findings in rodent models.

## 3. Discussion

The studies discussed in this review provide strong evidence that CGA modulates adipogenesis and lipid metabolism in both in vitro and in vivo animal models.

Several studies indicated that CGA treatment reduced adipocyte differentiation and inhibited lipid accumulation (Table 1), indicating anti-adipogenic effects. In vitro studies have reported a reduction in master adipogenic transcription factors (PPARγ and C/EBPα) and lipogenic enzyme expression (FAS, ACC, SREBP-1c), while in vivo studies report a reduction in triglyceride content and lipid accumulation. These findings collectively suggest a diminished capacity for adipogenic differentiation and lipid storage in adipocytes. Moreover, several in vitro studies reported the browning of white adipocytes and enhanced thermogenic gene levels, accompanied by elevated oxygen consumption and mitochondrial biogenesis, suggesting increased cellular respiration and thermogenic function. Similarly, in vivo studies reported reduced body weight, fat mass, adipocyte size, and hepatic lipid content in obese animals. Others reported a reduction in plasma pro-inflammatory cytokines and adipose tissue macrophage infiltration in animals fed high-fat diets or diabetic models.

Some studies demonstrated metabolic-sensitizing effects of CGA, indicated by improved glucose homeostasis and insulin sensitivity. Several in vitro studies reported that CGA increased glucose uptake and enhanced GLUT4 translocation in adipocytes, effects associated with dual PPARα/γ agonistic activity and improved insulin signaling (Table 1). In mature adipocytes, CGA treatment enhanced insulin-stimulated glucose uptake while reducing glucose incorporation into lipids. The evidence from the in vivo studies suggests improved insulin sensitivity, glucose tolerance, and reduced hyperinsulinemia following CGA supplementation, associated with reduced hepatic steatosis and improved pancreatic β-cell function. Additionally, several studies reported potent anti-inflammatory effects of CGA, demonstrated by suppression of pro-inflammatory cytokines (TNF-α, IL-6, IL-1β, MCP-1) and reduced NF-κB signaling in adipose tissue.

A few in vitro studies indicate that CGA activates AMPK signaling, while one suggested that the canonical Wnt/β-catenin pathway mediates anti-adipogenic effects through GSK-3β phosphorylation and β-catenin stabilization (Table 1) (Figure 2). The activation of AMPK by CGA led to phosphorylation of ACC, increased CPT1 expression, and enhanced fatty acid oxidation, while AMPK-dependent mechanisms mediated thermogenic activation in brown adipocytes. Additionally, CGA activated Shp2-mediated Erk1/2 signaling in human bone marrow-derived mesenchymal stem cells, which led to the suppression of adipogenic differentiation. This in vitro evidence aligns with some in vivo studies demonstrating AMPK-dependent metabolic improvements and increased fatty acid oxidation in adipose tissue of CGA-supplemented animals. Utilizing AMPK-specific and Wnt/β-catenin pathway knockdown approaches is necessary to fully elucidate the relative contribution of each signaling mechanism. The same approach can be applied to understand the interactions between multiple pathways activated by CGA.

Several studies employed CGA concentrations ranging from 5 to 200 µg/mL (approximately 14–560 µM) in vitro, with lower concentrations (5–50 µM) demonstrating significant anti-adipogenic and thermogenic effects.

The animal studies summarized in this review provide evidence that CGA administration may prevent diet-induced obesity, improve glucose homeostasis and insulin sensitivity, reduce hepatic steatosis, and attenuate inflammation in high-fat diet-fed rodents and diabetic models (Table 2) (Figure 3). CGA demonstrated efficacy in both preventive and therapeutic protocols, with db/db diabetic mice showing improvements in late-stage diabetes through adiponectin receptor signaling pathways. Additionally, CGA exhibited organ-protective effects by reducing renal fibrosis markers (AR, TGF-β1) in diabetic nephropathy and improving hepatic lipid dysregulation in endotoxin-challenged rats. However, clinical studies evaluating the anti-obesity effects of CGA in humans are currently lacking, representing a critical gap in the translation of preclinical findings into clinical applications.

## 4. Limitations and Future Directions

It is worth mentioning that the current body of evidence faces a consistent set of methodological and translational limitations that temper the interpretation of reported benefits. The following section outlines several key limitations and discusses how these issues are currently being addressed or may be mitigated in future research.

### 4.1. Supra-Physiological Concentration Use In Vitro

One of the more frequently flagged limitations in cell-based studies is the use of concentrations that far exceed what is physiologically achievable in human plasma. In human studies, peak plasma concentrations of polyphenols typically range between 0.1 and 10 µM after ingestion of polyphenol-rich foods, yet many in vitro experiments use CGA at 10–100 µM, while some utilize concentrations even higher (Table 1) [42,43]. This pharmacological-level dosing creates a disconnect between observed cell-based effects and what could realistically occur in vivo, making it difficult to directly extrapolate efficacy from 3T3-L1 or other adipocyte models to human dietary intake scenarios.

While this assessment remains true, it must be noted that the primary function of in vitro experiments is not to replicate systemic pharmacokinetics but to identify and characterize molecular targets and signaling nodes that are candidates for in vivo validation. Kerimi et al. (2005) articulate that, in the context of polyphenol research, in vitro studies provide an important early platform for identifying candidate mechanistic pathways, with in vivo models then serving to validate and extend those mechanistic insights under physiologically realistic conditions [44]. This two-stage paradigm—mechanistic mapping in vitro → pathway validation in vivo—is precisely why in vitro studies remain indispensable even when they rely on concentrations exceeding plasma achievability. For CGA specifically, pathways such as AMPK activation, PPARγ modulation, and NF-κB suppression identified at 10–100 µM in 3T3-L1 cells are confirmed to be active at lower exposures in HFD mouse and db/db mouse models, validating their translational relevance [34].

### 4.2. Bioavailability

Although chlorogenic acid (CGA) possesses considerable therapeutic potential, its clinical use is constrained by unfavourable pharmacokinetic characteristics, which often include hydrophilicity, rapid elimination, and mainly, low oral bioavailability. Mentioned below are a few key factors that limit CGA bioavailability.

#### 4.2.1. Extensive Biotransformation

Absorbed chlorogenic acid (CGA) may be directly utilized following absorption or undergo extensive biotransformation, including hydrolysis and subsequent conjugation with sulfate, glucuronic acid, or methyl groups [44]. Approximately one-third of ingested CGA is absorbed directly through the stomach and small intestine (ileum and jejunum) into systemic circulation, while the remaining fraction undergoes biotransformation by gut microbiota before absorption in the colon [45,46,47]. After absorption, CGA and its metabolites are further processed in the liver, where they are converted into biologically active metabolites such as caffeic acid and ferulic acid [48,49,50].

Furthermore, factors such as dietary composition and CGA concentration can significantly influence its absorption, bioavailability, and subsequent therapeutic effects [51,52,53,54,55]. It is also worth mentioning that a study performed by Gonthier et al. (2003) also indicates the gut microbiome may play a role in determining CGA bioavailability, although the evidence remains limited [56].

#### 4.2.2. Hydrophilicity

CGA’s low hydrophilicity is often attributed to its hydrophilic nature, which is justified by its Log P ≅ − 1.44 and six hydrogen bond donors (violating Lipinski’s Rule of Five), CGA making it too hydrophilic to efficiently cross the hydrophobic lipid bilayer of the intestinal epithelium via transcellular passive diffusion [57,58]. The small fraction of CGA that is absorbed primarily follows the paracellular route through tight junctions—a pathway with extremely limited surface area (~0.01% of total intestinal surface) and restrictive size cutoffs. Studies using Caco-2 monolayers confirm this, showing predominantly apical-side retention and very low transepithelial flux compared to more lipophilic metabolites like caffeic acid [59,60]. Compounding this, active efflux transporters (likely MRP2/BCRP) push absorbed CGA back into the intestinal lumen, further reducing net uptake [59,60,61].

To overcome the limitations of hydrophilicity and extensive biotransformation, various advanced drug delivery strategies have been explored to improve the bioavailability of CGA. Nanocarrier-based systems have attracted particular interest due to their ability to enhance targeted delivery while minimizing toxicity, thereby increasing the therapeutic effectiveness of CGA [62]. For example, researchers developed cyclodextrin (CD)-based formulations that improved the stability and intestinal absorption of CGA, highlighting CD complexation as an effective approach for enhancing its oral bioavailability [63]. In addition, nanophytovesicles (NPVs) have emerged as promising carriers for improving the physicochemical properties of plant-derived compounds. Trivedi et al. (2023) successfully optimized phospholipid-based NPVs for CGA delivery, resulting in enhanced solubility, permeability, and oral bioavailability [57]. Collectively, these findings demonstrate that innovative delivery platforms can substantially improve CGA bioavailability, supporting its potential for future clinical applications.

#### 4.2.3. Taste Receptors

Another mechanism that may be involved in the therapeutic effects of CGA is the activation of bitter taste receptors (T2Rs). Bitter taste receptors (T2Rs), a family of approximately 25 functional G protein-coupled receptors (GPCRs) that were initially characterized in oral taste buds but are now recognized to be widely expressed in numerous extraoral tissues [64]. Importantly, T2Rs are co-expressed on enteroendocrine cells, the specialized epithelial cells responsible for the secretion of gastrointestinal hormones. Multiple T2R subtypes, including T2R5, T2R14, T2R38, and T2R46, are co-localized with glucagon-like peptide-1 (GLP-1) in L-cells of both the small and large intestine, as demonstrated in human enteroendocrine cell lines (HuTu-80 and NCI-H716) and intestinal tissue biopsies [65]. Similarly, cholecystokinin (CCK)-secreting I-cells of the duodenum and jejunum express several T2R subtypes, including T2R14 and T2R38. This anatomical co-localization positions T2Rs as luminal chemosensors capable of translating the presence of unabsorbed dietary polyphenols into endocrine signaling responses [65,66].

The intracellular signaling cascade initiated by T2R activation has been well characterized. Binding of bitter ligands to T2Rs induces dissociation of the α-gustducin and Gβγ subunits, leading to activation of phospholipase Cβ_2_ and subsequent generation of inositol 1,4,5-trisphosphate (IP_3_) and diacylglycerol (DAG). This signaling cascade stimulates intracellular Ca^2+^ release and activates the TRPM5 ion channel, resulting in membrane depolarization and the exocytotic secretion of hormone-containing vesicles. Concurrently, the Gα-gustducin subunit activates phosphodiesterase, promoting cAMP degradation, while DAG and Ca^2+^ activate protein kinase C [64,65]. Collectively, these signaling events culminate in the release of preformed hormone granules from enteroendocrine cells, representing a luminal mechanism that does not require systemic absorption of the polyphenol.

Trius-Soler and Moreno (2024) identified numerous dietary polyphenols that function as ligands for T2Rs [65]. It is possible that CGA activates intestinal T2Rs, representing an additional mechanism that may contribute to its therapeutic effects. Although the existing evidence suggests that T2R-mediated signaling may constitute an important pathway through which CGA exerts its physiological actions, this hypothesis requires further experimental validation. Future studies should specifically investigate the role of intestinal T2Rs in mediating the metabolic and therapeutic effects of CGA.

### 4.3. Effect of Gut-Microbiota Modulation

In vivo effects of CGA are deeply intertwined with gut microbiota composition, since most CGA is metabolized colonically. The biotransformation rate of CGA by gut microbiota is lower in obese vs. lean individuals, and microbiota composition varies by diet, housing, and strain [66,67,68]. This means the anti-obesity effect observed in CGA-treated HFD mice may be substantially mediated by gut microbiota remodeling rather than direct adipocyte action, a confound that cannot be separated in most in vivo designs without germ-free. Therefore, additional investigation is needed to elucidate whether the effects of CGA on lipid metabolism are mediated by direct interactions with the gut microbiota, or whether the observed improvements in the gut microbial environment arise as a downstream consequence of its other beneficial physiological actions.

### 4.4. Use of Male-Biased Models In Vivo

As evident from this review, the majority of in vivo CGA studies use male rodents exclusively, ignoring well-documented sex differences in adipose tissue expansion, lipid metabolism, adipokine secretion, and HFD response [69,70,71,72]. We believe that future in vivo studies should investigate the potential sex-specific differences in the physiological response to CGA.

## 5. Conclusions

Overall, the scientific evidence currently available shows that CGA exhibits anti-obesity properties (Figure 2 and Figure 3). However, more thorough animal and human studies are required to investigate the anti-obesity potential of CGA in the context of human obesity and metabolic disorders in order to better understand the efficacy, safety, and therapeutic potential of this phytochemical.

## Figures and Tables

**Table 1:** Summary of effects of CGA and combination therapies on adipogenesis in vitro.

Study	Cell Line	Treatment	Effects
[23] (2015)	Human BMSCs	CGA 0.1, 1, 10, and 100 μM 14 days	↓ Lipid Accumulation ↓ PPARγ and C/EBPα mRNA ↑ p-Erk1/2
[24] (2017)	3T3-L1 Adipocytes And RINm5F cells	CGA 50 μM Pioglitazone 10 μM	150% ↑ in PPARγ mRNA 40% ↑ in PPARα mRNA 25% ↑ in FATP mRNA
[25] (2019)	Mouse C_3_H_10_T_1/2_ Embryonic Fibroblasts	CGA 20, 30, and 50 μM 6 days	→ PPARα, CPT1α, ATGL, HSL, ACS, C/EBPα, PPARγ2, and PRDM16 → Lipid droplet accumulation ↑ UCP1 mRNA ↑ Glucose Uptake ↑ GLUT2 and PFK ↑ TFAM, ATP5A, UQCRC2, SDHB, and p-AMPK protein
[26] (2021)	3T3-L1 Adipocytes	CGA 50, 100, 200 μg/mL 12 Days	→ Cell viability loss for up to 240 μg/mL ↓ Lipid accumulation ↓ PPAR-γ, aP2, FASN, and LPL mRNA and protein ↑ Wnt10b and β- catenin ↓ t-GSK-3β ↑ p-GSK-3β
[27] (2026)	3T3L1 Adipocytes	CGA 5–20 µM H_2_O_2_ 100µM	↓ SA-β-Gal, IL-6, IL-8, TNF-α, MMP-3, and COX-2 ↑ PPAR-γ, FASN, PI3K, AKT, GLUT4

Table legend: ↑: increased, ↓: reduced, →: unchanged, t: total, p: phosphorylated, CGA: Chlorogenic Acid, SA-β-Gal: Senescence-associated β-galactosidase, CM: Conditioned Media, PPAR-γ: Peroxisome Proliferator-Activated Receptor-γ, CPT1α: Carnitine Palmitoyl Transferase 1, HSL: Hormone-sensitive Lipase, ATGL: Adipose Triglyceride Lipase, UCP-1: Uncoupling Protein 1, aP2: Adipocyte Protein 2, FASN: Fatty-Acid Synthase, LPL: Lipoprotein Lipase.

**Table 2:** Summary of effects of CGA and combination therapies on adipogenesis in vivo.

Study	Animal Model	Treatment	Effects
[28] (2010)	ICR Male Mice	CGA 0.2% (*w*/*w*) 8 weeks HFD (37% fat, 21% beef tallow)	↓ Body weight and Weight gain ↓ eWAT, plasma TG, Total cholesterol ↑ HDL-c: Total Cholesterol ratio ↓ FASN, HMG-CoA reductase, ACAT ↑ β-oxidation, PPARα
[29] (2015)	C57BL/6J Male Mice	CGA 100mg/kg bi-weekly 15 weeks HFD (60% kcal fat) _________________ CGA 100mg/kg bi-weekly 6 weeks (HFD 60% kcal fat)	↓ Body weight gain, Adiposity ↓ eWAT and BAT Hypertrophy ↓ F4/80, CD68, CD11b, CD11c ↓ TNFα, MCP-1, CCR2 ↓ PPARγ, CD36, FABP4, MGAT1 ↑ CPT1a, CPT1b, PPARα, ACOX1, FGF21 ↑ Insulin sensitivity ↓ Fasting blood glucose, Insulin ↑ Glucose clearance ______________________________ → Body weight gain, Adiposity ↑ Insulin sensitivity ↓ Fasting blood glucose, Insulin ↑ Glucose clearance
[30] (2015)	C57BL/BKS db/db Female Mice	CGA 80 mg/kg/day 12 weeks	↓ % VAT, Fasting plasma glucose, HbA1c ↑ Adiponectin ↓ TGF-β1 ↑ ADPNR-2, p-AMPK, G-6-P, PPAR-α, ADPNR-1, GLUT-4
[31] (2016)	Sprague-Dawley Female Rats	CGA 60 mg/kg/day 28 days	↓ Weight gain, VAT weight ↓ adipocyte area, serum TG, and FFA ↑ HDL-c ↓ Serum bilirubin, ALT, and AST ↑ β-oxidation, and CPT-1 ↓ t-ACC ↑ p-AMPK
[32] (2019)	C57BL/6J Male Mice	CGA 150 mg/kg/day 6 weeks HFD (18.4% kcal fat)	↓ Body weight, Weight gain, liver weight, and eWAT ↓ Fat mass and Adipocyte size ↓ Lipid droplet accumulation ↓ Total Cholesterol, plasma TG, LDL-c ↑ HDL-c, plasma ALT, AST, BUN ↓ PPAR-γ, C/EBP-α, SREBP-1c, FAS, LPL, AP2, and GRP43 ↑ PPARα and Adiponectin
[33] (2021)	C57BL/6 Male Mice	CGA 15mg/kg/day 20 weeks HFD	↓ Body weight gain ↓ Inguinal, perirenal, and eWAT ↓ Fasting blood glucose, Fasting insulin ↑ Insulin Sensitivity, ↓ Glucose Tolerance ↓ plasma LPS, TNF-α, IL-1β, and MCP-1, TLR-4 ↑ Intestinal abundance of *Dubosiella*, *Romboutsia*, *Mucispirillum*, *Faecalibaculum* genus bacteria.
[34] (2022)	db/db Male Mice db/m Male Mice	CGA 250 mg/kg/day 18 days	↓ Body weight, weight gain, inguinal fat weight ↑ Glucose tolerance and Insulin sensitivity ↓ Fasting blood glucose ↓ Serum TG, Total cholesterol, LDL-c ↑ HDL-c ↓ ALT, AST, Lipid accumulation ↑ CPT1a, ACOX1, ATGL, HSL, IL-10, SOD1, SOD2, GPX1 ↓MGAT1, DGAT1, DGAT2, CD36, FATP4 ↓ TNF-α, IL-1β, IL-6
[35] (2025)	Sprague-Dawley Male Rats	CGA 10, 100 mg/kg/day 4 weeks HFD (60% kcal from lard)	↓ Weight Gain, BMI, Abdominal Circumference, and Abdominal WAT mass ↓ Serum Insulin, Fasting Blood Glucose, Leptin ↑ Adiponectin ↓ Total Cholesterol, TG levels, LDL-c ↑ HDL-c ↓ IRAK1 and TRAF6, TNF-α, NF-κB p65, IL-1β, and IFN-γ ↓ p53, Bax, and Caspase-3 ↑ NRF2, HO1, SOD, CAT, and GPx

Table legend: ↑: increased, ↓: reduced, →: unchanged, t: total, p: phosphorylated, CGA: Chlorogenic Acid, *w*/*w*: weight/weight, HFD: high-fat diet, BMI: Body Mass Index, WAT: White Adipose Tissue, TG: Triglyceride, LDL-c: Low-Density Lipoprotein-cholesterol, HDL-c: High-Density Lipoprotein-cholesterol, SREBP-1c: Sterol Regulatory Element-Binding Protein-1c, VAT—Visceral Adipose Tissue.

**Table 3:** Summary of effects of combination therapies in vitro and in vivo.

Study	Model	Treatment	Effects
[36] (2020)	Human SGBS Adipocytes	CGA + CA 5, 10, and 50 μM 9 days	↑ AMPK mRNA ↑ UCP1 and PGC1α ↓ ACC, FASN, and SREBP1 ↓C/EBPα, and PPARγ
[37] (2021)	3T3-L1 Adipocytes	CGA + CF 40 μg/mL, 160 μg/mL 12 days	↑ AMPK, ACO, CAT, ATGL, GLUT4, HSL mRNA ↓ GPDH mRNA ↓ PPAR-γ protein ↓ C/EBP-α protein
[38] (2019)	ICR Female Mice	CGA + CF 0.2% + 0.02%, 0.2% + 0.04% (*w*/*w*) 14 weeks HFD (45% kcal fat)	↓ Serum total cholesterol, TG, LDL-c, Leptin, IL-6 ↓ Hepatic TG and Cholesterol ↑ ACO, and HMGR mRNA ↓ FAS, SCD1, SREBP-1c, and LXRα mRNA ↑ p-AMPKα, ATGL, and HSL mRNA and protein ↓ FAS, SCD1, and LXRα
[39] (2023)	C57BL/6 Male Mice	CGA + MET 0.25% (*w*/*w*) 10 weeks HFD	↑ Arginase and CD206 ↓ iNOS and CD11c ↑ IL-10 ↓ TNF-α, IL-1β, MCP-1, and IL-6 ↓ Fasting blood glucose, Glucose tolerance

Table legend: ↑: increased, ↓: reduced, p: phosphorylated, CGA: Chlorogenic Acid, CF: Caffeine, CA: Caffeic Acid, MET: Metformin, *w*/*w*: weight/weight, HFD: high-fat diet, TG: Triglyceride, SREBP-1c: Sterol Regulatory Element-Binding Protein-1c, GPDH: Glycerol-3-phosphate dehydrogenase.

**Table 4:** Summary of effects of CGA in human studies.

Study	Subjects	Treatment	Effects
[40] (2007)	Humans 6 Males, 6 Females Mean Age 24.2 ± 3.2 years BMI < 25 kg/m^2^ ______________ Humans (n = 30) BMI 27.5–32.0 kg/m^2^	Coffee Slender^®^ (Orally) _______________ Coffee Slender^®^ (11g/day) 12 weeks	↓ Plasma Glucose ↓ Body Weight ↓ Body Fat %
[41] (2013)	Humans Male Mean Age 36.1 ± 7.4 years	CGA (325mg/day) 4 weeks	→ Body Weight, BMI, Body Fat ratio ↑ Post-Prandial Energy Expenditure → Carbohydrate Utilization ↑ Fat Utilization

Table legend: ↑: increased, ↓: reduced, →no change.

## Data Availability

No new data were created or analyzed in this study. Data sharing is not applicable to this article.
